# Evaluation of SUV normalized by lean body mass (SUL) in ^68^Ga-PSMA11 PET/CT: a bi-centric analysis

**DOI:** 10.1186/s13550-019-0572-z

**Published:** 2019-12-02

**Authors:** Andrei Gafita, Jeremie Calais, Charlott Franz, Isabel Rauscher, Hui Wang, Andrew Roberstson, Johannes Czernin, Wolfgang A. Weber, Matthias Eiber

**Affiliations:** 1 Department of Nuclear Medicine, Technical University of Munich, Klinikum rechts der Isar, Munich, Germany; 2 Department of Molecular and Medical Pharmacology, David Geffen School of Medicine at UCLA, Los Angeles, USA

**Keywords:** SUL, PSMA PET, SUV

## Abstract

**Introduction:**

The aim of this analysis was to investigate whether the standardized uptake value (SUV) normalized by lean body mass (SUL) is a more appropriate quantitative parameter compared to the commonly used SUV normalized by patient’s weight in ^68^Ga-PSMA11 PET/CT.

**Material and methods:**

^68^Ga-PSMA11 PET/CT scans of 121 patients with prostate cancer from two institutions were evaluated. Liver SUV was measured within a 3-cm volume-of-interest (VOI) in the right hepatic lobe and corrected for lean body mass using the Janmahasatian formula. SUV and SUL repeatability between baseline and follow-up scans of the same patients were assessed.

**Results:**

SUV was significantly positively correlated with body weight (*r* = 0.35, *p* = 0.02). In contrast, SUL was not correlated with body weight (*r* = 0.23, *p* = 0.07). No significant differences were found between baseline and follow-up scan (*p* = 0.52).

**Conclusion:**

The Janmahasatian formula annuls the positive correlations between SUV and body weight, suggesting that SUL is preferable to SUV for quantitative analyses of ^68^Ga-PSMA11 PET/CT scans.

## Introduction

In the last decade, positron emission tomography/computer tomography (PET/CT) has gradually emerged as the standard-of-care imaging modality in the diagnosis and treatment response monitoring of different oncological malignancies [1, 2]. Moreover, standardized uptake value (SUV), which is the commonly used quantitative parameter in PET/CT showed a high predictive value for treatment outcome [3, 4]. However, quantitative SUV is still hampered by a number of physiological, technical and physical factors widely discussed in the literature [5].

In ^18^F-FDG PET/CT, SUV normalized by patient’s weight is known to be highly dependent on body weight [6]. Since ^18^F-FDG does not significantly accumulate in adipose tissue in the fasting state, the use of SUV can falsely lead to high values in patients with high body mass. Subsequently, SUL (lean body mass (LBM)–corrected SUV) has been proposed as a more appropriate quantitative method, with Janmahasatian formulation for LBM showing most accurate results [7].

$$ LBM=\frac{9.27\times {10}^3\times BW}{6.68\times {10}^3+216\times BMI} $$

The liver is typically used as reference organ in PET imaging, with a 3-cm spherical volume of interest (VOI) computed to measure the liver background activity [8]. In ^18^F-FDG PET/CT, liver SUL showed only a fair repeatability between two time points in the same patient [9].

In the past 5 years, prostate-specific membrane antigen (PSMA), a transmembrane protein highly expressed in prostate cancer, has become a promising target for PET in prostate cancer imaging [10]. ^68^Ga-PSMA11 PET/CT has shown enhanced accuracy compared to conventional imaging modalities in lesion detection [11], with SUV being largely used as a quantitative PET-derived parameter [12, 13]. However, the effect of body weight on SUV in ^68^Ga-PSMA11 PET/CT has not been yet investigated. Since ^68^Ga-PSMA11 does not typically accumulate in adipose tissue [14], we hypothesized that liver SUV is dependent from body weight.

The aim of the present study was to investigate whether SUL is a more appropriate quantitative method compared to the commonly used SUV normalized by body weight in ^68^Ga-PSMA11 PET/CT imaging.

## Material and methods

### Patients

Patients from two institutions, Technical University Munich (COH1) and University of California Los Angeles (COH2), who underwent ^68^Ga-PSMA11 PET/CT prior to ^177^Lu-PSMA radioligand therapy were included. Patients in whom it was not feasible to draw a 3-cm VOI in healthy liver tissue were excluded.

For COH1, 91 subsequent patients who received between October 2014 and March 2018 were considered for this analysis. For COH2, 43 subsequent patients were prospectively enrolled in a phase 2 trial (NCT03515577). All patients signed a written consent for evaluation of their data and the institutional review board of the Technical University Munich (permit 5665/13) and University of California Los Angeles (permit 17-000330) approved this analysis.

### Image acquisition

Images were obtained in accordance with the international guideline [15] in conjunction with a diagnostic following application of ^68^Ga-PSMA-11 that was synthesized as described previously [16]. The ^68^Ga-PSMA-ligand complex solution was applied to patients via an intravenous bolus with a mean of 146.0 ± 45.4 and 192.3 ± 19.7 MBq for COH1 and COH2, respectively. PET acquisition was started at a mean time of 66.5 ± 12.8 and 56.4 ± 9.7 min after tracer injection for COH1 and COH2. The PET was reconstructed by ordered subset expectation maximization (OSEM)-based algorithms. Data from the CT scan were used for attenuation correction.

### Image analyses

Images were reviewed using qPSMA, an in-house developed software [17]. Body weights were recorded from the patients’ records.

#### SUV and SUL

For liver SUV computation, the VOI was semi-automatically placed using an algorithm [18] that has shown excellent intra- and inter-reader agreement. SUL was calculated according to the Janmahasatian formula as follows:

$$ SUL= SUV\times \frac{LBM}{BW} $$

#### Repeatability

To assess repeatability and the potential influence of tumor sink effect two ^68^Ga-PSMA11 PET/CT scans from a subset of patients at two different dates were included in the analysis. SUV and SUL of both scans were compared.

### Statistical analysis

Values were reported as mean ± SD. Pearson correlations were performed to evaluate the relationship between SUV, SUL, and body weights. Paired t-test was used when the values were considered as paired. *p* value < 0.05 was considered statistically significant. Analyses were performed using SPSS Statistics v22.0 (IBM Corp., USA).

## Results

### Patient cohort

In total, 121 patients were included in the final analyses. Eighty patients of COH1 were eligible; as in 11 patients, the 3-cm VOI in the liver was not feasible due to severe breathing artifacts. Forty-one patients of COH2 could be included as two patients underwent ^18^F-DCFPyL PET/CT prior to ^177^LuPSMA treatment. Notably, significantly higher activity doses were injected for COH2 (mean ± SD,118 ± 25 vs. 192 ± 19 MBq, *p* < 0.001). Mean ± SD of body weight were 80.3 ± 11.4, 82.5 ± 17.5 and 81.0 ± 13.7 kg for COH1, COH2, and all patients, respectively. Patient characteristics are presented in Table 1.

**Table 1:** Patients characteristics

	All patients (*n* = 121)
Age (years)	73 ± 7.3
Body weight (kg)	81.0 ± 13.7
Time since diagnosis of prostate cancer (years)	7 ± 11
Gleason score at diagnosis^a^	
< 8	38 (36%)
≥ 8	69 (64%)
PSA at the time of PET/CT imaging (ng/ml)	114 ± 671
Prior lines of systemic treatment	
2	8 (7%)
≥ 3	113 (93%)
≥ 4	78 (64%)
≥ 5	44 (36%)
≥6	25 (21%)
Sites of disease on PSMA-PET	
Bone	112 (93%)
Lymph nodes	97 (80%)
Visceral^b^	43 (36%)
Bone + lymph nodes	88 (73%)
Bone + lymph nodes + visceral	34 (28%)

Data are median ± standard deviation or *n* (%)

*PSA*, prostate-specific antigen; *PSMA*, prostate-specific membrane antigen

^a^Data missing for 14 patients

^b^Visceral includes liver, lungs, and adrenal glands

### SUV, SUL, and comparisons

Mean ± SD of liver SUV were 4.30 ± 1.55, 4.58 ± 1.57, and 4.36 ± 1.56 for COH1, COH2, and all patients, respectively. Mean ± SD of liver SUL were 3.23 ± 1.16, 3.41 ± 1.03, and 3.29 ± 1.12 for COH1, COH2, and all patients, respectively. Both cohorts did not show any significant differences for body weights (*p* = 0.46), SUVs (*p* = 0.27), or SULs (*p* = 0.37).

**Fig. 1:**
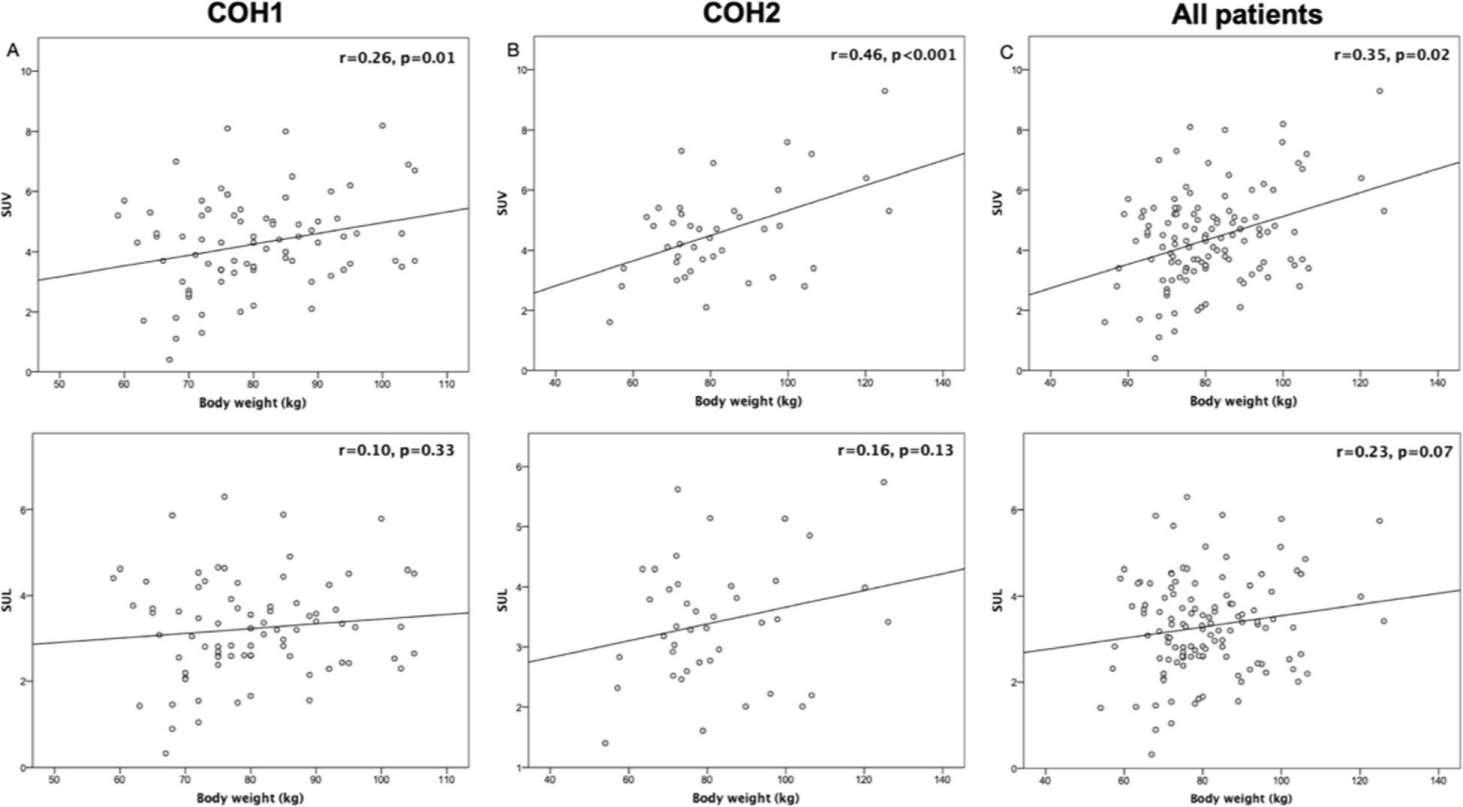
Scatter plots of both SUV and SUL correlations with body weight for **a** COH1, **b** COH2, and **c** all patients

SUV showed a significant, but weak correlation with body weight (*r* = 0.26, *p* = 0.01), (*r* = 0.46, *p* < 0.001), and (*r* = 0.35, *p* = 0.02) for COH1, COH2, and all patients, respectively. In contrast, SUL was not significantly correlated with body weight (*r* = 0.10, *p* = 0.33), (*r* = 0.16, *p* = 0.13), and (*r* = 0.23, *p* = 0.07) for COH1, COH2, and all patients, respectively. Scatter plots of correlations for both SUV and SUL with body weight are displayed in Fig. 1.

### Repeatability

Sixty patients from COH1 received a follow-up ^68^Ga-PSMA11 PET/CT during ^177^Lu-PSMA radioligand therapy at a mean ± SD of 3.7 ± 0.6 months after the baseline scan. Mean ± SD-injected dose for baseline and follow-up scan was 118 ± 25 and 105 ± 23 MBq, respectively.

**Fig. 2:**
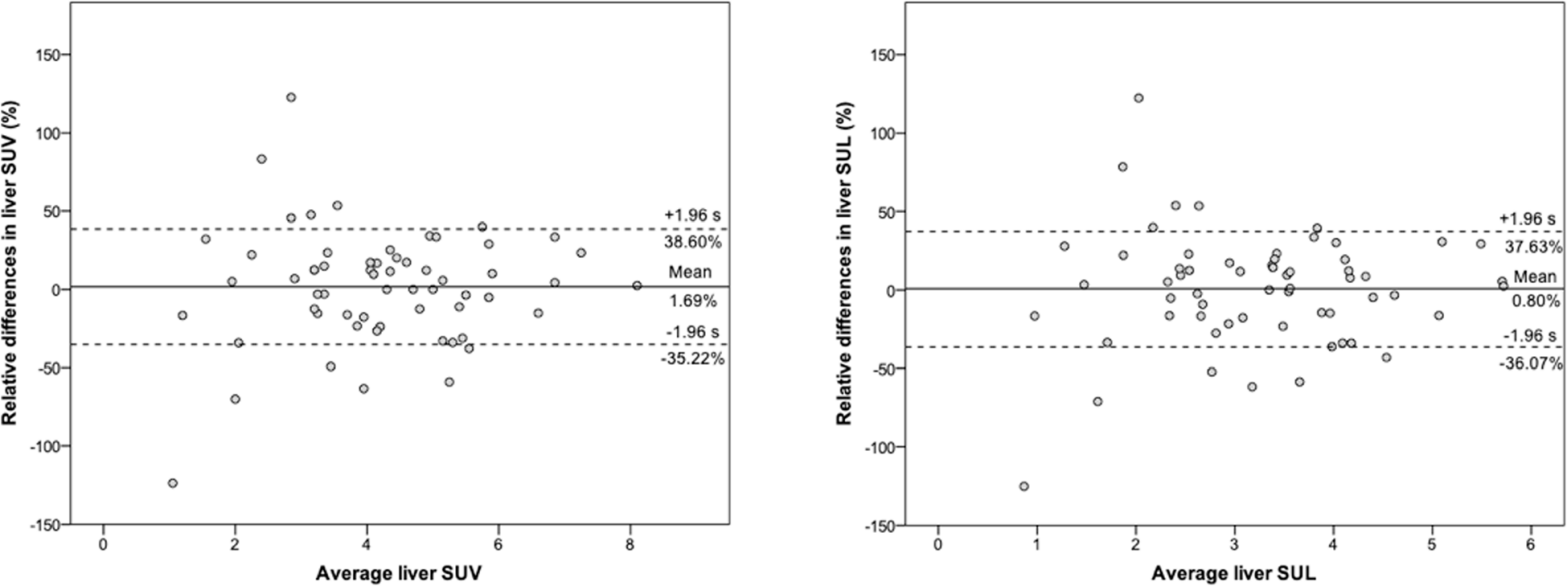
Bland-Altman plots of baseline and follow-up ^68^Ga-PSMA11 PET/CTs agreement for both liver SUV and SUL

Mean liver SUV did not change significantly (*p* = 0.52) between the baseline (4.26 ± 1.64) and follow-up scan (4.16 ± 1.60). Mean (95%CI) relative difference was 1.69 (−7.84;11.22)% with ICC of 0.821 (0.701–0.893). Mean liver SUL did not change significantly (*p* = 0.72) between the baseline (3.25 ± 1.22) and follow-up scans (3.21 ± 1.22). Mean (95% CI) relative difference was 0.80 (−8.70; 10.32)%, with ICC of 0.818 (0.696–0.892). Figure 2 displays the Bland-Altman plots for SUV and SUL.

## Discussion

To the best of our knowledge, this is the first report evaluating the potential of using SUL as compared to the commonly used SUV as quantitative parameter in ^68^Ga-PSMA11 PET/CT. Our data indicate that a weak but significant positive correlation is present between liver SUV and body weight. Contrarily, SUL as alternative parameter seems to be unaffected from body weight. Since ^68^Ga-PSMA11 does not typically accumulate in adipose tissue the use of SUL can be recommended to avoid any influence from patient habitus.

PET-derived parameters, such as SUV_mean_ or SUV_max_, are increasingly used for therapy response monitoring or patient outcome prediction. Therefore, highly accurate computed parameters should be addressed given their potential decisive role for the clinical image–based decisions. The present study attempted to reproduce a clinical setting where quantitative ^68^Ga-PSMA11 PET/CT scans are used in the framework of ^177^Lu-PSMA radioligand therapy.

We investigated the relation of body weight with SUV and SUL in a patient cohort including subcohorts of both European and North-American patients. Consistently among the subgroups, our findings indicate that liver SUL calculated based on Janmahasatian formula annuls the body weight dependence of liver SUV. Noteworthy, even though a significant correlation was found between body weight and SUV, its strength is rather weak (*r* = 0.35, *p* = 0.02). However, since SUL (*r* = 0.23, *p* = 0.07) annuls and lowers the strength of the positive correlations to BW, the use of SUL should be preferred over SUV. In FDG PET/CT, liver SUV showed a moderate correlation for women (*r* = 0.58, *p* < 0.001) and men (*r* = 0.54, *p* < 0.001) with body weight, which was annulled and reduced by the Janmahasatian formula, respectively [7].

In addition, we have to stress that the high interpatient repeatability of both liver SUV and SUL between two time points (ICC = 0.821 and 0.818) demonstrates an acceptable mean difference of 1.69% and 0.80%. Despite potential changes of liver ^68^Ga-PSMA11 uptake during ^177^Lu-PSMA therapy upon shifts of biodistribution depending on tumor sink effect, the use of liver as the reference organ to threshold malignancy comparing subsequent timepoints is feasible [19]. Notably, it has been shown that only high differences in tumor burden have significant implications on liver ^68^Ga-PSMA11-uptake, with low vs. high tumor load exhibiting a liver SUV_mean_ of 4.34 vs. 3.27, *p* < 0.001 [13].

Androgen deprivation therapy (ADT) has shown to increase the PSMA-ligand uptake in the first weeks after treatment initiation in metastatic sensitive prostate-cancer [20]. Moreover, continuous long-term ADT significantly decreased lesion uptake in ^68^Ga-PSMA11 PET imaging [21]. However, these findings might not be valid for patients with metastatic castration-resistant prostate cancer, since most of their tumor lesions are not responding properly to first-line ADT. No significant differences were noted between liver SUV in patients receiving ADT versus not receiving [22]. Same analysis further evaluated the ^68^Ga-PSMA11 uptake of other tissues such blood pool (SUV_mean_ 1.08) or muscle (SUV_mean_ 0.50). Nevertheless, the liver showed the most feasible values (SUV_mean_ 4.73) to be used for PSMA PET quantification. The mean liver SUV obtained in the present analysis (4.36) is in concordance with those obtained by Jansen et al (4.73) and Gaertner et al. (4.25) [13].

Interestingly, in an analysis including 64 patients who received ^18^F-DCFPyL PET/CT no correlations were found between both liver SUV and SUL with body weight [23]. Comparing the results, for ^18^F-DCFPyL were obtained higher liver SUV and SUL values: 5.1 ± 0.7 vs. 4.4 ± 1.5 and 3.8 ± 0.5 vs. 3.3 ± 1.1 respectively. Similar to FDG, PSMA-ligands do not typically accumulate in adipose tissue, therefore a positive correlation between SUV and BW annulated by SUL was expected to be found for both radiopharmaceuticals.

For PSMA-targeted radioligand therapies, ^68^GaPSMA11 PET imaging is typically used at baseline for patient selection, as well as during treatment for radiographic response assessment. However, the clinical utility of ^68^GaPSMA11 PET in metastatic castration-resistant prostate cancer goes beyond the radioligand therapy, being increasingly used for evaluation of treatment response in patients receiving, e.g., taxanes [24]. Since the traditional SUV has shown a significant correlation with body weight, its clinical value in ^68^Ga-PSMA11 PET quantification remains questionable. Thus, our findings may have clinical implications especially in treatment response assessment. However, further studies comparing the prognostic value of both SUV and SUL for imaging response evaluation are warranted.

Notable limitations of the current analysis are the retrospective nature of the study and the inclusion of a selected patient cohort scheduled for ^177^Lu-PSMA radioligand therapy. However, as our patients have shown both high and low tumor load the potential influence of different tumor burden is already acknowledged. Finally, our analysis is focused on the liver as most important normal organ in PSMA-ligand PET imaging severing as reference tissue. We have not investigated the influence of body weight on other normal tissues.

## Conclusion

Our results indicate that the Janmahasatian formula annuls the positive correlations between absolute SUV and body weight, suggesting that SUL is preferable to SUV for quantitative analyses in ^68^Ga-PSMA11 PET. Future studies, are necessary to determine the clinical significance of the differences between SUV and SUL for different clinical applications, such as thresholds for delineation of tumor volumes or monitoring tumor response to therapy.

## Data Availability

Please contact the corresponding author for data requests.

## Abbreviations

ADT: Androgen deprivation therapy
BW: Body weight
LBM: Lean body mass
PET: Positron emission tomography
PET/CT: Positron emission tomography/computer tomography imaging
PSMA: Prostate-specific membrane antigen
SUL: Standardized uptake value normalized to lean body mass
SUV: Standard uptake value
VOI: Volume of interest
